# Evaluation of the Queensland JEV vaccine program response to the 2022 Australian outbreak

**DOI:** 10.1017/S0950268824001730

**Published:** 2024-12-20

**Authors:** Angus Misan, Stephen B. Lambert, Hai Phung, Megan K. Young

**Affiliations:** 1 School of Medicine and Dentistry, Griffith University, Gold Coast, Queensland, Australia; 2 Communicable Diseases Branch, Queensland Health, Brisbane, Queensland, Australia; 3 Metro North Health, National Centre for Immunisation Research and Surveillance, Westmead, New South Wales, Australia; 4 Sydney Children’s Hospital Network, Metro North Public Health Unit, Brisbane, Queensland, Australia; 5 School of Public Health, University of Queensland, Brisbane, Queensland, Australia

**Keywords:** geographic distribution, Japanese encephalitis, One Health, vaccination (immunization), vaccine program

## Abstract

In 2022, the largest ever virgin soil outbreak of Japanese encephalitis (JE) occurred in Australia resulting in 45 reported human cases of JE, with seven fatalities. Japanese encephalitis virus (JEV) was detected in 84 piggeries across Australia. In response, states implemented targeted vaccination programs for those individuals at the highest risk of JEV exposure. A mixed methods approach, including geospatial mapping of JEV vaccine distribution in Queensland, a case series of Queensland human cases and interviews with Queensland Health staff, assessed the JEV vaccination response program. Five notified human cases were reviewed, with three having occupational outdoor risk and local travel-related exposure. Vaccine coverage ranged from 0 to 7.4 doses per 100 people after 12 months of the program. The highest uptake was in southern Queensland, where 95% of the state’s commercial pig population is located. The vaccination program was limited by a heavy reliance on general practitioners, vast geographical distribution of eligible populations, difficulties mobilising and engaging eligible cohorts, and suboptimal One Health collaboration. Population and climate factors make it possible for the virus to become endemic. Targeted vaccination programs remain an important strategy to protect people at the highest risk of exposure, however, program improvements are required to optimize vaccine accessibility.

## Introduction

Japanese encephalitis virus (JEV) is a single-stranded RNA flavivirus transmitted from pigs and water birds to humans by a mosquito vector [1]. JEV is endemic in 24 countries in Asia and the Western Pacific [2] and causes approximately 68,000 cases globally annually [3]. JEV is notifiable in Australia and prior to 2021 was rare, with the only locally acquired human cases recorded (*n* = 5) occurring in far northern Queensland in 1995 and 1998, caused by JEV genotype GII.

In 2022, the detection of locally acquired JEV genotype GIV was notified in three Australian states for the first time in history, representing a significant change in the virus’ presence in Australia. This followed the detection of a sentinel human case in February 2021 in the Tiwi Islands, Northern Territory [4]. Phylogeographic analysis suggests GIV was limited to Indonesia and Vietnam from 1980 and spread to the Tiwi Islands between 2005–2020, and then finally spread to Queensland between 2020–2022 [5]. On 4 March 2022, the resulting human outbreak was declared a Communicable Disease Incident of National Significance (CDINS) [6]. During the outbreak, 45 human cases and 84 infected piggeries were detected nationally, of which 5 human cases and 18 piggeries were in Queensland [7–10].

The potential endemicity of JEV in Australia is of considerable concern due to the impact of reproductive losses in the commercial pig industry, and the potential severe human health consequences for those who develop clinical Japanese encephalitis (JE) [11]. Although <1% of people who contract JEV develop JE, the virus has a 20–30% case fatality rate in individuals who develop acute encephalitis, and of the survivors, 30–50% have long-term neurological sequelae [3].

The role of One Health collaboration and consideration of the One Health triad (human, animal and environmental health) in a JEV outbreak response is of great importance. One Health is an integrated and unified approach acknowledging the connections between humans, animals and the environment to sustainably optimize the health of all, through collaboration between multiple sectors, disciplines and communities [12]. Given the complex zoonotic transmission cycle and environmental factors that directly impact mosquito abundance, a true One Health approach is essential for an effective JEV response and control program. Sharing of information between all stakeholders in an outbreak is essential to adopting an effective vaccination program that identifies high-risk individuals through surveillance of vectors and animal hosts.

Given the complex interplay of vectors, hosts, and environment, and the unknown risk factors for acquiring JEV in Australia, several control measures were implemented. These included mosquito management in piggeries, enhanced mosquito surveillance, public health messaging about mosquito avoidance behaviours, and a human vaccination program (JEV vaccines are not registered for use in pigs in Australia). The rollout of the vaccination program was an essential component of the outbreak response. JEV vaccines available in Australia are Imojev, a live attenuated vaccine that is registered for people aged 9 months and older, and JEspect, an inactivated vaccine that is registered for those aged 2 months and older, requiring two doses given one month apart [13]. The Communicable Diseases Network Australia (CDNA) determined recommendations for eligibility criteria for vaccination, declaring those with direct exposure or close proximity to pigs and mosquitoes and high-level occupational exposure priority groups for vaccination [14]. This eligibility criteria aligns with global guidance from the World Health Organization (WHO), which recommends targeting populations with exposure to animal reservoirs and ecological conditions supportive of virus transmission [15]. Queensland Health followed these guidelines, prioritizing those who had close proximity to or worked with pigs and mosquitoes [16].

Of all Australian states, Queensland has the largest commercial pig herd (27% of the national herd) and the most abundant feral pig population [17, 18]. An abundance of animal hosts, vectors, suitable tropical and temperate climates, and proximity to endemic areas of South-East Asia, increase the likelihood of endemicity in Queensland and future JEV outbreaks. This study aimed to contribute to future JEV control strategies in Queensland by evaluating the state’s targeted vaccination program. This included assessing possible risk factors for acquiring JE in cases, JEV vaccination uptake, the geospatial distribution of vaccinated residents relative to the commercial pig industry, and the application of a One Health approach.

## Methods

### Study design

This descriptive study included: a case series detailing the clinical and demographic information of five human JE cases in Queensland; a geospatial distribution of human JEV vaccinations in Queensland over two phases of vaccine eligibility; and interviews with Queensland Health staff about the vaccination program. Phase one vaccine eligibility commenced on 1 March 2022 and included people who:

1. worked at, lived near, or had a planned, non-deferable visit to a piggery, pork abattoir (or pork rendering plant), including farm workers and their families living at the piggery, transport workers, veterinarians and others involved in the care of pigs;
2. personnel who worked directly with mosquitoes through their surveillance (field or laboratory-based) or control and management such as environmental health officers and workers and entomologists; and
3. diagnostic and research laboratory workers who may be exposed to the virus.

**Figure 1.**
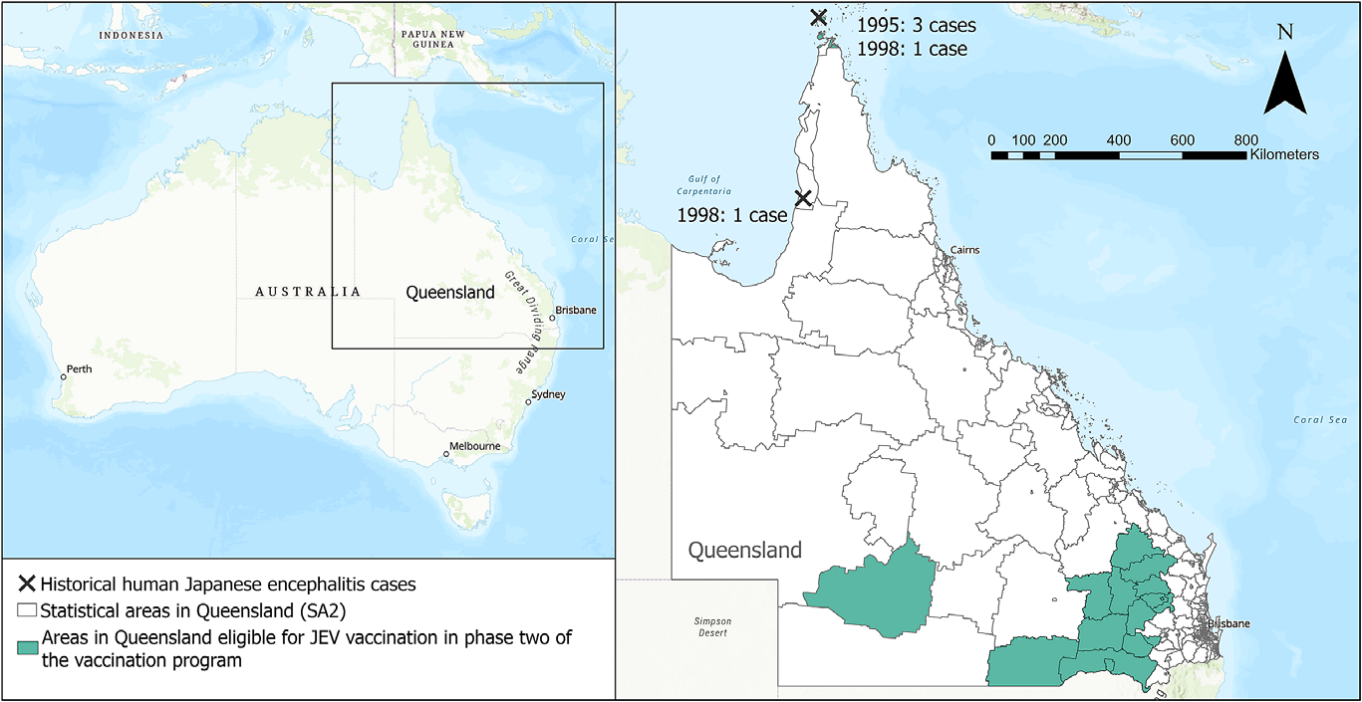
The statistical areas (SA2) in Queensland eligible for JEV vaccination during the Queensland Health vaccination response program, between 12 September 2022–28 February 2023, and historic, locally acquired cases of Japanese encephalitis in Australia (3 in 1995, 2 in 1998). The eligible SA2s make up the LGAs of Balonne, Goondiwindi, North Burnett, South Burnett, Western Downs, Quilpie and Southwest Toowoomba, in addition to the Torres Strait which was eligible for routine vaccination prior to the outbreak..

Phase two commenced on 12 September 2022 and extended phase one eligibility to include people who lived or worked in specific Local Government Areas (LGAs) and spent occupational or recreational time outdoors performing activities near potentially productive mosquito habitats, such as rivers, ponds, and marshes (Figure 1) [16].

### Data collection

De-identified routinely collected data on laboratory-confirmed or probable cases of JE in Queensland, as defined by the Communicable Disease Network Australia [19], notified 1 January 2022–28 February 2023 were accessed from Queensland Health’s Notifiable Conditions System (NOCS) [20].

JEV vaccination records for Queensland residents, administered between 1 March 2022 and 28 February 2023, were extracted from the Australian Immunization Register (AIR) [21]. Extracted data included total daily vaccinations administered in Queensland by vaccine brand, age groups, sex, and the Statistical Area (SA2) of residence, as registered with Medicare, Australia’s universal healthcare insurance scheme. SA2s are medium-sized (3,000–25,000 people) general-purpose areas used to examine population data.

Two semi-structured group interviews were conducted with a total of nine representatives of Queensland Health’s Communicable Diseases Branch, Queensland Health’s Immunization Program and Darling Downs Public Health Unit. Participants were purposively recruited for interviews (performed by one author (AM)), given their knowledge of and involvement in the planning and rollout of the JEV vaccination program. Interviews, conducted in September and October 2023, were transcribed using Microsoft Teams software (ver 1.6.0). Logistics, strengths, weaknesses and the One Health approach of the JEV response, including the vaccination program, were the focus of the interviews.

Estimates of the commercial pig populations across SA2s in Queensland were obtained from the 2021 Australian Bureau of Statistics (ABS) Agriculture Commodities data [22]. The human population data across SA2s in Queensland were obtained from the 2021 ABS Regional Population estimates [23]. Locations and detection dates of positive JEV detections in pigs (LGA) are provided (personal communication, Dr. Joanne Mollinger, Manager Projects, Department of Agriculture and Fisheries). Positive mosquito locations were previously reported [24], and co-ordinates of vaccine service providers (VSPs) were obtained from the Queensland Health website [25].

### Data analysis

Descriptive case series data for five human cases of JE were reported. Daily and monthly totals of vaccine doses administered across SA2s in Queensland were calculated from extracted AIR data. AIR data were assigned to either phase one (1 March 2022–11 September 2022) or phase two (12 September 2022–28 February 2023) by date of administration. Chi-squared analyses were performed using IBM SPSS Statistics software (version 29.0.0) to compare the demographics of recipients across phases. Human vaccine doses/10,000 pigs for each SA2 with >1,000 pigs were calculated by dividing AIR phase one dose totals by commercial pig population ABS data. To examine vaccination coverage after 12 months of the vaccination program, the number of vaccine doses/100 people was calculated for each SA2 by dividing AIR 12-month vaccination totals by human population ABS data. Transcripts from interviews were de-identified and analyzed by one investigator (AM). Themes were identified within the following topics: logistics of the rollout, strengths and weaknesses of the vaccination program, and level of One Health collaboration.

#### Geospatial distribution of vaccine doses

Choropleth maps were generated using ArcGIS Pro software (ver 2.8.0) by spatially joining data files to SA2 shape files, to examine the distribution of vaccine doses administered across southern Queensland for different phases of vaccine eligibility. In phase one, the total number of vaccine doses administered and registered on the AIR was mapped for each SA2. Additionally, LGAs with JEV-positive piggery detections (the number of detections was displayed as a centroid within the LGA), the commercial pig population, the SA2s with positive mosquito detections, and likely locations where human cases acquired JEV (displayed as a centroid within the SA2) were mapped. In phase one, for each SA2 with >1,000 commercial pigs, the number of vaccine doses/10,000 commercial pigs was mapped to give a relative vaccination rate and hence an estimate of the vaccine uptake in the piggery workforce for each SA2. The total number of vaccine doses administered and registered on the AIR in the first 12 months of the response was mapped, in addition to LGAs eligible for vaccination and the location of VSPs within and surrounding eligible LGAs. The total number of vaccine doses/100 people after 12 months of the program was also mapped.

### Ethical considerations

The study was approved by the Metro North Human Research Ethics Committee (HREC). A waiver of consent for NOCS data and AIR data was approved by Metro North HREC and data were de-identified before becoming accessible to project investigators. Written informed consent was provided by participants prior to interviews.

## Results

**Figure 2.**
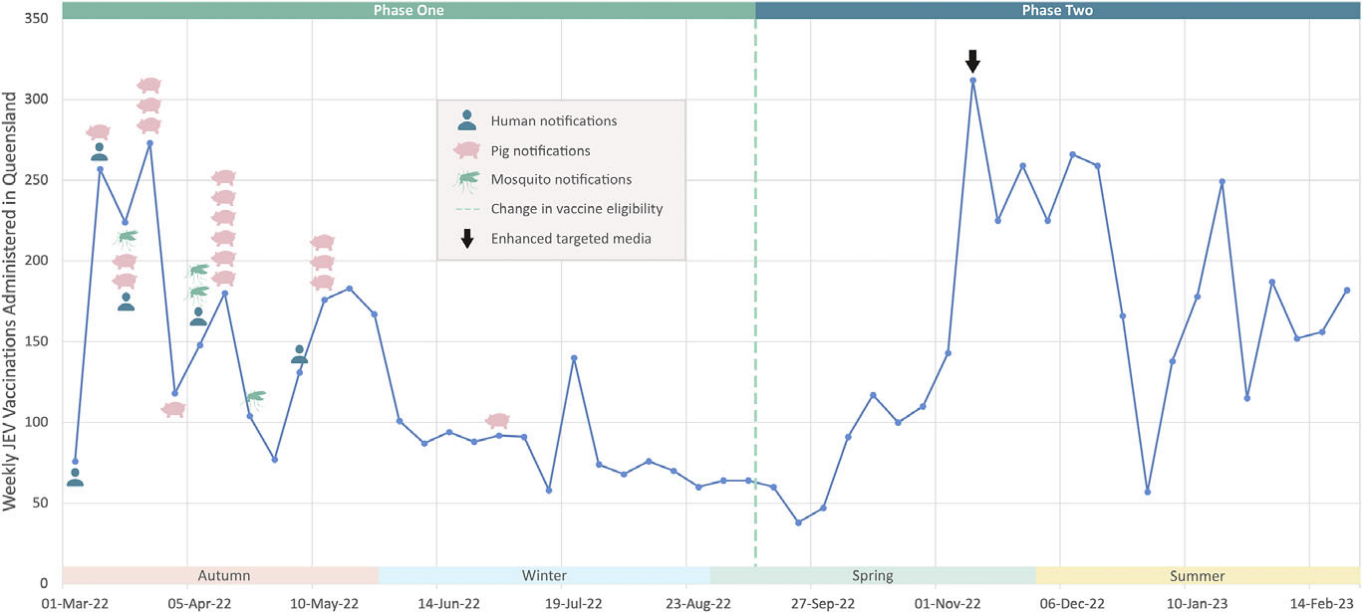
Total weekly vaccine doses administered and registered on the Australian Immunisation Register during the first 12 months of the Queensland Japanese encephalitis vaccination response program from 1 March 2022–28 February 2023 (n = 7,185 doses), showing notifications of positive JEV detections in humans, pigs and mosquitoes during that time. .

The five Queensland cases of JE were notified between March–May 2022 (Table 1). No further cases were notified in Queensland as of 11 November 2024. Three of the cases worked in the farming and agricultural industry, one was a teacher, and the other was retired. Three cases had travelled through LGAs with the detection of JEV in piggeries. None of the five cases were JEV vaccinated, and none had travelled outside of Australia during their exposure periods. Following the CDINS declaration on 4 March 2022 there were notifications of pig and mosquito JEV detections in Queensland from March–July 2022 (Figure 2). Over the first 12 months of the vaccination response, a total of 7,185 JEV vaccine doses were administered in Queensland and registered on the AIR. Weekly vaccine doses administered increased at the beginning of the response then reduced over winter, before peaking in late spring and continuing to remain high during the summer. The peak in November coincided with increased media engagement, particularly targeted in the Darling Downs area, where 47% of vaccine doses in Queensland were administered between 8–14^th^ November 2022.

**Table 1.** Case series of human JEV cases notified during the outbreak and reported to the Notifiable Conditions System, including demographics, symptoms, exposure details, laboratory results, and outcome if known

Case number	1	2	3	4	5
Onset date	Dec 2021	Jan 2022	Jan 2022	Feb 2022	Mar 2022
Patient	46-year-old man	59-year-old woman	36-year-old woman	68-year-old woman	22-year-old woman
Status	Probable^d^	Probable	Confirmed	Confirmed	Probable
Symptoms	Headache Muscle ache Shortness of breath Fatigue Stomach cramps	Fever Headache Muscle ache Backache Fatigue Itch Abnormal taste Nausea Chills Orbital pain Confusion Agitation Convulsion Disorientation Jaundice Areflexia	Fever Headache Photophobia Confusion Seizures Disorientation Convulsions	Fever Headache Rash Lethargy Nausea Diarrhoea Confusion Disorientation	Headache Muscle ache Joint ache Lethargy Nausea Diarrhoea Confusion Weakness Chest pain
Encephalitis	Unknown	Yes	Yes	Yes	Unknown
Exposure period	Nov 2021	Jan 2022	Jan 2022	Feb 2022	Feb–Mar 2022
Exposure period travel locations	Quilpie Maidenwell Dalby	Toowoomba Miles Dalby Jandowae Chinchilla	No travel	Millmerran Tara Chinchilla	Goondiwindi
Serology	**Blood**: IgG positive^a^, IgM positive^b^	**CSF**: IgM positive^b^	**Blood**: IgG positive^a^, IgM positive^b^ **CSF**: IgM positive^c^	**Blood**: IgG positive^a^ (seroconversion, initially IgG non-reactive) IgM positive^b^ **CSF**: IgM positive^b^	**Blood**: IgG positive^a^, IgM positive^b^
Hospitalization	Unknown	Yes	Yes	Yes	No
Outcome	Survived	Died	Survived	Survived	Survived

a Flavivirus IgG MIA - Reactive.

b Flavivirus (serotype) IgM MIA – Japanese Encephalitis Virus.

c Flavivirus (serotype) IgM MIA – Flavivirus.

d This case was epidemiologically linked to another case, and in combination with serology, an assessment was made that this illness was consistent with the male being a probable case.

### Demographics of vaccine recipients

In the first month of the vaccination program 882 vaccines were administered, with one-third of those administered to children aged 0–9 years, and 53% to those <30 years (Supplementary Figure 1). During phase one, 45% of vaccines were administered to people <30 years, compared to 31% during phase two. Children aged 0–9 years made up a higher proportion of those vaccinated during phase one compared to phase two (22.3% vs. 9.4%; *p* < 0.001) (Table 2).

**Table 2.** JEV vaccine doses administered and registered on the Australian Immunization Register during phase one (1 March 2022–11 September 2022) (*n* = 3,333) and phase two (12 September 2022–28 February 2023) (*n* = 3,852) eligibility periods by sex, vaccine type and age group of recipients

Variable	Phase 1 *N* = 3,333 *n* (%)	Phase 2 *N* = 3,852 *n* (%)	*P*-value
Sex			
Female	1,327 (39.8)	1867 (48.5)	<0.001
Male	2006 (60.2)	1985 (51.5)	<0.001
Vaccine type			
Imojev	3,038 (91.1)	3,387 (87.9)	<0.001
JEspect	97 (2.9)	325 (8.4)	<0.001
Other^a^	198 (5.9)	140 (3.6)	<0.001
Age group			
0–9 years	744 (22.3)	363 (9.4)	<0.001
10–19 years	177 (5.3)	246 (6.4)	0.053
20–29 years	591 (17.7)	586 (15.2)	0.004
30–39 years	538 (16.1)	545 (14.1)	0.019
40–49 years	518 (15.5)	483 (12.5)	<0.001
50–59 years	410 (12.3)	610 (15.8)	<0.001
60–69 years	278 (8.3)	592 (15.4)	<0.001
70–79 years	57 (1.7)	324 (8.4)	<0.001
80–89 years	16 (0.5)	94 (2.4)	<0.001
90–99 years	4 (0.1)	9 (0.2)	0.258

a ‘Other’ includes AIR JEV vaccine doses recorded as Generic Japanese Encephalitis (4.0% of total vaccine doses), JE-VAX and 0.1 ml intradermal (<1.0% of total vaccine doses).

### Geospatial distribution of JEV vaccine doses

**Figure 3.**
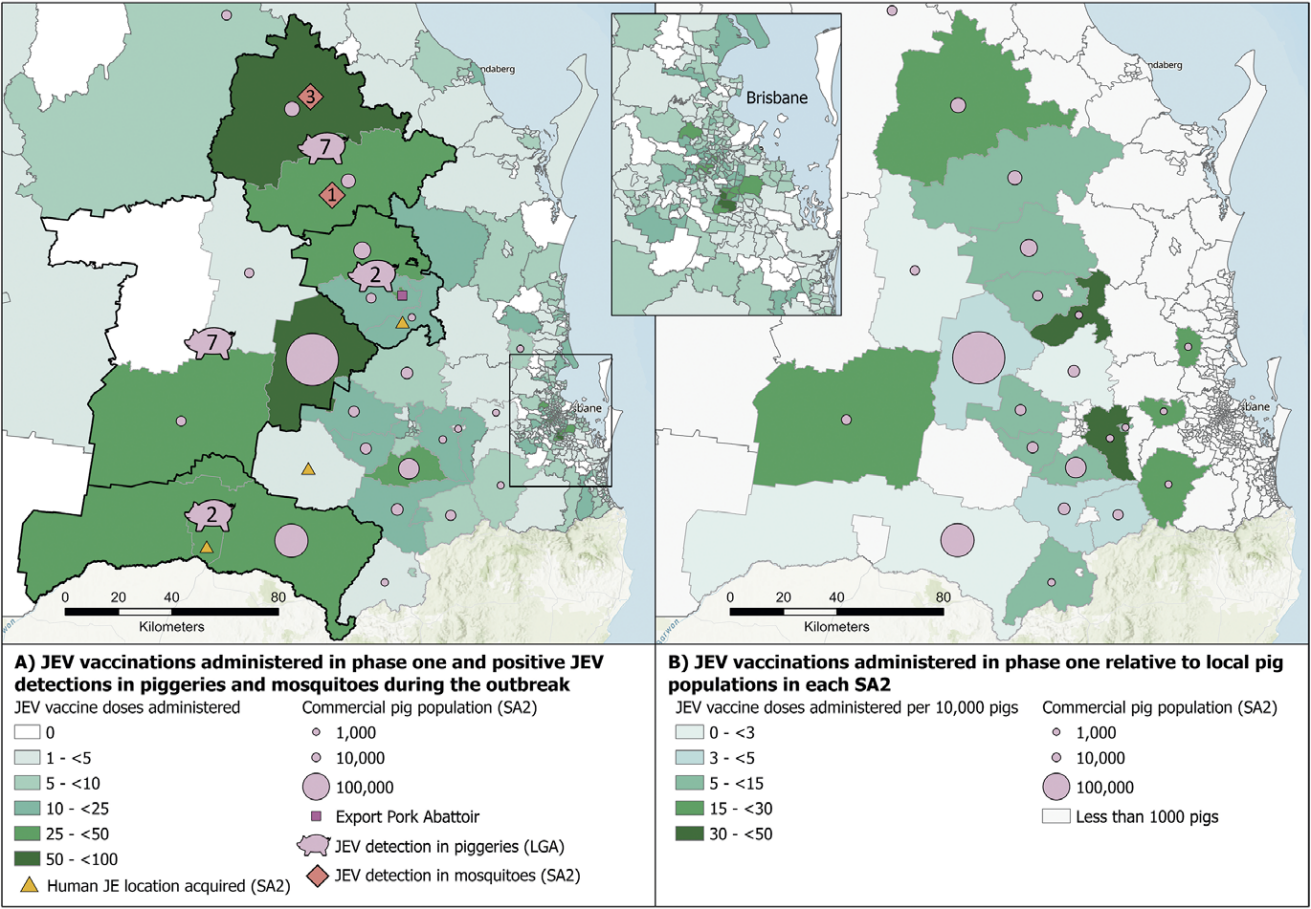
JEV vaccine doses administered and registered on the Australian Immunisation Register during phase one (1 March 2022–11 September 2022) of the Queensland Japanese encephalitis vaccination response program (n = 3,333 doses): A) the total number of vaccine doses administered, the commercial pig population (SA2), Queensland’s export pork abattoir, locations where JEV was detected in mosquitoes (SA2), in the 18 piggeries (LGAs) and the most likely location where three of the five human JE cases acquired JEV infection (SA2); and B) relative rate of vaccine doses administered, taking into consideration the commercial pig population as a representation of the commercial pig industry workforce (SA2). .

**Figure 4.**
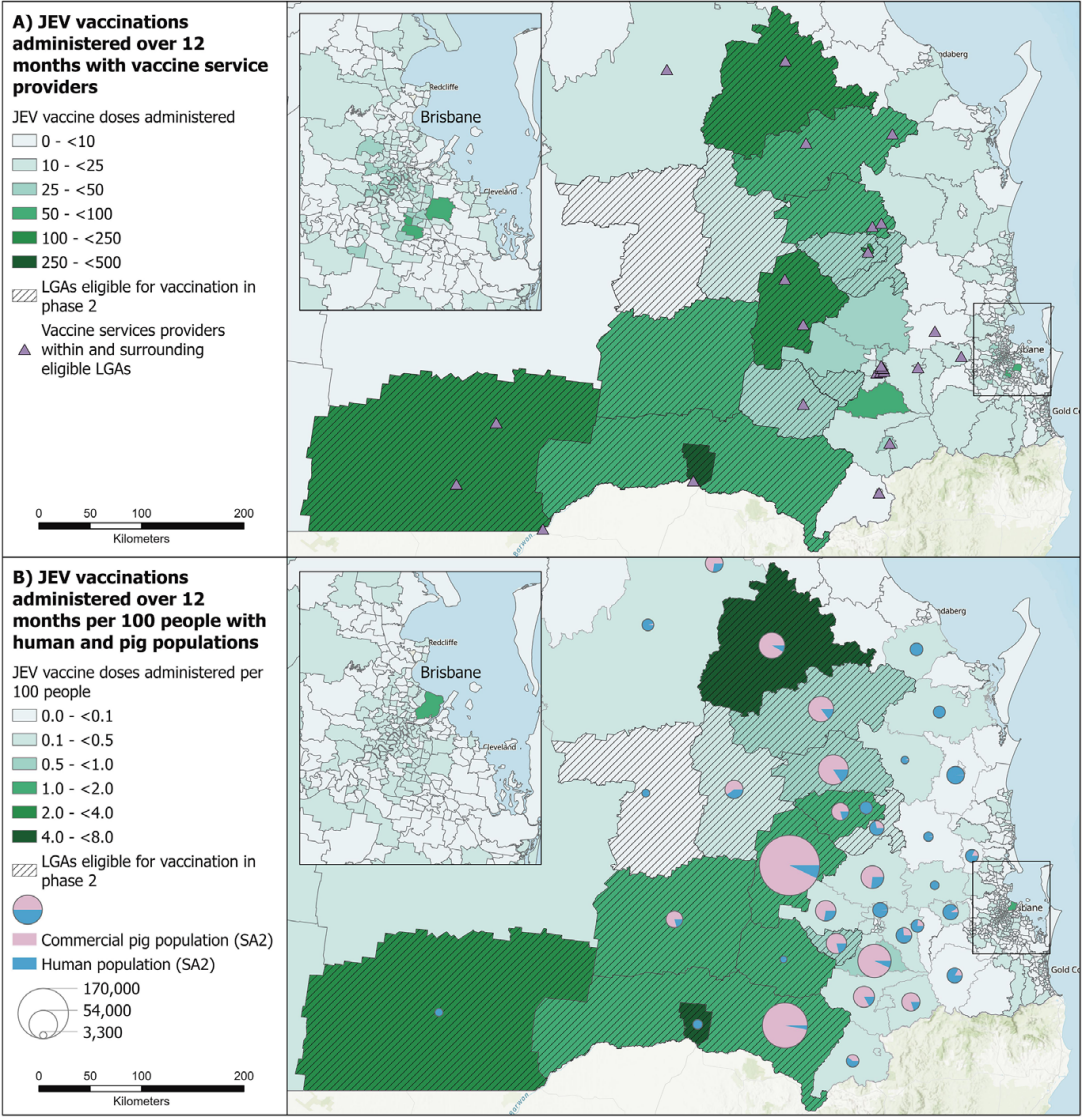
JEV vaccine doses administered and registered on the Australian Immunisation Register during the first 12 months of the Queensland Japanese encephalitis vaccination response program, from 1 March 2022–28 February 2023 (n = 7,185 doses) including the specific LGAs that were eligible during this period; Balonne, Goondiwindi, North Burnett, South Burnett, Western Downs and Southwest Toowoomba: A) total number of vaccine doses administered for each SA2 and the location of vaccine service providers administering JEV vaccines within and surrounding eligible LGAs; and B) number of vaccine doses administered per 100 people as an estimation of vaccine coverage, includes the local human and commercial pig populations for each SA2. .

In phase one, 3,333 vaccines were administered across Queensland. During phase one, the number of vaccinations administered varied between SA2s, with the highest uptake in southern Queensland where approximately 95% of the commercial pig herd is located (Figure 3). In SA2s with >1,000 commercial pigs, the number of doses administered/10,000 pigs ranged from <3 to ≥30. In phase one, vaccine uptake was highest in Wambo (93 doses) and Monto-Eidsvold (79 doses). Wambo has the largest pig population in Queensland (~225,000 pigs), and Monto-Eidsvold had three of Queensland’s four JEV-positive mosquito detections. After 12 months, the highest uptake remained in southern Queensland where all but one of the LGAs of eligibility were located (Figure 4). The highest vaccine coverage was in the SA2s of Goondiwindi and Monto-Eidsvold with 7.4 and 5.5 doses/100 people, respectively.

### Interviews about vaccination program rollout

#### Logistics of the rollout

Initially, Queensland Health procured 600 doses of Imojev and JEspect, in addition to their existing supply for the routine vaccination program in the Torres Strait Islands. Overall, just over 34,000 doses were eventually received, with 5,000 doses arriving in each of April, June, and August, and a further 17,000 doses in September 2022. Vaccine doses were distributed to a range of VSPs, including general practices (GPs, Australia’s primary care physicians), Aboriginal community-controlled health services, community health clinics, hospitals, and council clinics.

#### Gaps in the vaccination program

The program weakness of greatest significance, identified by interview participants, was that only medical practitioners were able to administer the JEV vaccine for the first 12 months of the program. Nurse-led vaccine delivery under the Extended Practice Authority (EPA) in Queensland only allowed nurses to administer the JEV vaccine in the Torres Strait and Cape York, as part of the already established vaccination program in this region of Queensland. Despite advocacy for amendments to the EPA to be expedited, it took 12 months before nurses were able to administer the JEV vaccine.

*Because of the nursing EPA, it had to be administered by a medical officer, or with their permission, which severely hampered the rollout of this vaccine.* [Health services manager, Queensland Health].

The request that GPs not charge a co-payment for vaccine administration without some form of financial recompense from the government was a barrier to enrolling them as VSPs. While some public health units and medical practitioners co-ordinated site vaccinations for areas with large numbers of eligible individuals, this settings-based approach was used infrequently, due to associated complexities, including expenses. Despite interest from private companies to be involved in the vaccine rollout, without timely amendments to the EPA, their nurses were not permitted to supplement the vaccine workforce. Participants also identified issues relying on GPs to administer vaccines in future outbreaks, particularly if a large number of doses needed to be administered quickly, and alternative methods of vaccine distribution and administration needed to be considered.

…*there is a need for surge vaccination capacity within health services… Now, health services would need to be resourced to do this…we have lent on GPs…and it’s simply unsustainable to keep doing so…So, we are having big barriers here* [Health services manager, Queensland Health].

Another key barrier to vaccine uptake articulated was limited vaccine access. Some cohorts eligible for vaccination lived in very remote areas and had to travel hours to VSPs, and non-Medicare-eligible individuals working in the pig industry would be required to pay clinic fees, deterring them from seeking vaccination. Lingering impacts of COVID-19, including unavailability of GP appointments and vaccine fatigue were also barriers. A significant challenge was onboarding VSPs, given that VSPs had to agree to store and administer JEV vaccines to eligible members of the public at no cost to the individual.

*The environment for general practice is very challenging financially at the moment…They are often booked out a couple of weeks in advance and the approach we have taken is to ask them to please distribute vaccines for us and not charge a fee, that is problematic.* [Health services manager, Queensland Health].

Access to reliable information about which eligible cohorts were receiving the vaccine doses was also a challenge. Queensland Health relied on AIR data, which lacked demographic details and relevant eligibility criteria for vaccine recipients. Participants also reported difficulties in prioritizing strategic communications. Despite targeted communications and social media campaigns when the vaccine supply increased, the increase in demand was modest.

#### Strengths of the vaccination program

Collaboration with VSPs was considered a strength, despite several challenges. Given the large geographical distribution of eligible populations, having a wide distribution of VSPs in regional and remote areas was essential. Access to reserve doses in hospitals in remote settings allowed for quicker re-distribution, to accommodate fluctuations in demand across Queensland. This local approach in rural and remote areas and the dedication from public health staff despite significant challenges was a strength of the response. Liaising and collaboration with pork industry bodies improved vaccine uptake, ensuring workers were engaged and vaccinated in a timely manner.

*In the piggeries, the companies were paying [if there was a co-payment] for their employees to be vaccinated. They were organizing transport to VSPs for them to get vaccinated so that certainly helped the good uptake in that cohort.* [Registered nurse, Queensland Health].

#### One Health collaboration

At the start of the outbreak, there were limitations in the level of collaboration and information sharing between human and animal health sectors. Biosecurity Queensland (responsible for animal surveillance) was unable to disclose the location of piggeries where JEV was detected, but rather provided regional locations, to maintain business anonymity. This likely impacted the accuracy of the placement of mosquito traps, potentially hindering surveillance efforts.

*We’d get a very vague area or LGA, but they [Biosecurity Queensland] would not give us an address… because they were very concerned about the commercial impacts on the pork industry… it really did impact how we could respond effectively from a mosquito surveillance perspective.* [Registered nurse, Queensland Health].

## Discussion

These study findings contribute to future JEV control strategies in Queensland by examining geospatial variability in vaccine uptake, strengths and weaknesses of the JEV vaccination program, and One Health collaborations during the first 12 months of the program. Of the 125,000 vaccine doses administered nationally in the first 16 months of the response [8] only 7,185 of those were administered and registered on the AIR in Queensland during the first year of the response.

The geospatial distribution of vaccine uptake, both in absolute terms and relative to human populations, was highest in southern Queensland (where ~95% of the commercial pig population is located [17]) and LGA locations that became eligible in phase two of the program. There was considerable spatial variability in uptake during phase one. The SA2 with the highest vaccine uptake had the largest commercial pig population in Queensland (~225,000 pigs). The second-highest SA2 in phase one had three of the four positive mosquito detections and is within the LGA with JEV detections in seven piggeries. There were several barriers that possibly influenced uptake in the first cohort of eligibility, including limited vaccine supply and VSPs. The highest vaccine coverage after 12 months was in an SA2 surrounded by large commercial piggeries, including two piggeries testing positive for JEV in February–March 2022 [26] and a human case was also likely acquired in this area. This may have motivated people in the area to seek vaccination and may explain the relatively high uptake. The vaccination peak in November 2022 coincided with increased media engagement in the Darling Downs area [27]. This highlights the importance of enhanced local media and the use of champions, such as the Chief Health Officer who visited the region during this time, to boost vaccination in target areas.

There was a notable difference in the age distribution of vaccines across the phases of the program. In phase one compared to phase two, more children aged <9 years were vaccinated (22.3% vs. 9.4%), while less adults aged >60 years were vaccinated (10.6% vs. 26.2%). Young children are more likely than adults to have severe neurological sequela and die from JEV. While this may have impacted parental decisions to vaccinate their children, the most likely explanation for the higher uptake of the vaccine among children in phase one is that piggery workers and farmers included a high proportion of people with young families who were also eligible for the vaccine at that time. Similarly, more adults aged >60 years would have become eligible in phase two which targeted individuals based on location, not only on occupational risk.

One of the weaknesses of the vaccination program was the reliance on GPs and medical officers to administer or oversee the administration of vaccines. In Australia, there are fewer GPs per 100,000 people in regional compared to metropolitan areas, compounded by reduced and variable workforce capacity [28]. Often in regional settings, GP clinics are relied upon because of a lack of other health providers. The size and remoteness of some eligible communities made it difficult to manage vaccine distribution and administration. This may have been a more significant issue in Queensland compared to some other states with smaller geographical and more densely populated regional and urban areas and a larger number of existing health provision services in those areas.

The environment for GPs post-COVID-19, during the JEV vaccine rollout, was financially challenging [29]. Relying heavily on GPs to store and administer vaccines with no co-payments likely negatively impacted the scale and timeliness of vaccine rollout. If an upscaled vaccination program was required in response to another JEV outbreak or similar public health emergency, a different GP payment model may be required alongside increasing workforce capacity. Use of settings-based vaccine administration after outbreaks, may counteract any immobility issues and ensure appropriate uptake for high-risk cohorts. A data recording system appropriate for primary care or as an adjunct to the AIR should be prioritized to collect demographic information and eligibility criteria.

A collaborative and multi-disciplinary approach between animal, human, and environmental health departments is essential to the detection and monitoring of JEV transmission in all jurisdictions and across all levels of government in Australia, and to adequately respond to future outbreaks of zoonotic diseases [30]. Despite One Health engagement in Queensland, the issue of timely and comprehensive data sharing from animal health with human health agencies was identified by a number of interviewees as an issue of concern. In the Queensland response, failure to share freely the locations of infected piggeries with the health department likely hampered mosquito surveillance and the targeted provision of vaccines in the early stages of the program. The lack of detailed information sharing with health authorities was also noted as an issue in the interim report by the Queensland Government [31], and also in the Joint National JEV Outbreak Response Plan, noting “a data sharing agreement” was under development between animal and human health agencies at a state, territory, and national level to formalize information sharing in future JEV responses [30].

Currently, the Australian government is establishing an Australian Centre for Disease Control (CDC), which commenced operations on 1 January 2024, to improve Australia’s response and preparedness for public health emergencies such as the JEV outbreak [32]. The Australian CDC will employ a One Health framework to bring resources and expertise together to tackle shared threats to human, animal, and environmental health to transcend the traditional barriers that exist between departments and organizations [12]. A One Health approach to surveillance and data sharing in Queensland is essential, given the large commercial pig industry and significant feral pig population.

### Potential future vaccination programs

It is well established that vaccination is an important tool in JE prevention [2, 3, 33–35]. When the distribution of JE is not uniform, targeted vaccination programs can be a more cost-effective method of prevention than population-wide programs [36, 37]. Targeted vaccination programs implemented following outbreaks can be successful [38]. As with the response to the 2022 JEV outbreak, future targeted vaccination programs must consider eligibility criteria based on evidence of risk factors. Some of the possible risk factors for the five human cases in Queensland included occupational risk from working outdoors, and extensive travel around regional areas where there was evidence of JEV transmission. It is acknowledged that the five detected cases in Queensland in this outbreak are likely an under-representation of the true rate of infection. Human surveillance relies mostly on pathology testing, and given most infections with JEV are asymptomatic, many individuals who have been infected are unlikely to be identified by the surveillance system. If localised transmission occurs in the future in Queensland, then some regions may implement population-based vaccination programs, much like the established JEV vaccination program in the Torres Strait Islands. The sustainability of such an approach will require a stable supply of vaccines.

### Study limitations

There are limitations in the vaccination data analysis in this study largely due to limitations within the datasets. True rates of vaccine coverage could not be calculated as AIR data did not easily identify primary or subsequent doses of JEspect. Therefore, the number of doses administered/100 people was used as an estimate of coverage. AIR data also contained vaccine types other than Imojev (89.4%) and JEspect (5.9%), including Generic Japanese Encephalitis (GJE) (4.0%), JE-VAX and 0.1 ml intradermal (<1.0%). The specific type of vaccine recorded as GJE and JE-VAX is unknown, and likely was either selected in error or the brand was unknown. However, these accounted for <5% of the total doses registered on the AIR, so were unlikely to impact coverage estimates. AIR data were aggregated to an SA2 level based on primary residential address, but some individuals may have worked or travelled in another SA2, impacting the interpretation of the geospatial distribution. Another limitation of this study was the inability to determine a denominator that accurately reflects the population at risk. In both phases of vaccination, the number of eligible people could not be measured. Therefore, the number of commercial pigs and the human population within each SA2 were used as denominators to estimate a comparative coverage in the piggery workforce and the total population respectively across SA2s. This study was also limited by the human health interview focus, and while there was consideration of the One Health approach to the outbreak, perspectives from animal health, animal industry, and environmental health have only briefly been explored.

## Conclusion

Eradication of JEV in Australia does not appear immediately feasible, however temporary elimination may have been achieved. Optimising the rollout of vaccination programs for humans is an essential component of JEV prevention. Geospatial mapping can assist with identifying high-risk locations and populations for active surveillance and prevention including prioritising vaccine distribution. To improve vaccine accessibility in future programs, a particular focus should be on the expansion of VSPs to include nurses and other service providers and exploring the use of alternative reimbursement models for GPs administering vaccines as part of a broad public health response. It will be important for Queensland to continue to pursue formal information-sharing agreements between the human and animal health authorities. Collaboration between human, animal and environmental authorities on operational activities including joint field investigations, data sharing, surveillance, vector control coordination and community engagement should be a priority for future responses. The establishment of the Australian CDC should assist in formalising and embedding a One Health approach into the management of future outbreaks and epidemics including JEV.

## Supporting information

Misan et al. supplementary material Misan et al. supplementary material

## Data Availability

Deidentified data about notified cases are available for request using standard approaches through Queensland Health. Data are not available for further distribution due to privacy issues.

## Supplementary material

For supplementary material accompanying this paper visit http://doi.org/10.1017/S0950268824001730. click here to view supplementary material
