# Evaluation of the Queensland JEV vaccine program response to the 2022 Australian outbreak – CORRIGENDUM

**DOI:** 10.1017/S0950268825000019

**Published:** 2025-01-17

**Authors:** Angus Misan, Stephen B. Lambert, Hai Phung, Megan K. Young

**Affiliations:** 1 School of Medicine and Dentistry, Griffith University, Gold Coast, Queensland, Australia; 2 Communicable Diseases Branch, Queensland Health, Brisbane, Queensland, Australia; 3 National Centre for Immunisation Research and Surveillance, Sydney Children’s Hospital Network, Westmead, New South Wales, Australia; 4 Metro North Public Health Unit, Metro North Health, Brisbane, Queensland, Australia; 5 School of Public Health, University of Queensland, Brisbane, Queensland, Australia

When this article was originally published in Epidemiology and Infection it contained an error in the affiliations 3 and 4. These have now been corrected and updated.

The authors apologise for this error.
