# Supplementary figures and images for: Evaluation of the Queensland JEV vaccine program response to the 2022 Australian outbreak
Source: Epidemiol Infect. 2024 Dec 20;152:e176. doi: 10.1017/S0950268824001730 (PMC11696590; doi:10.1017/S0950268824001730)

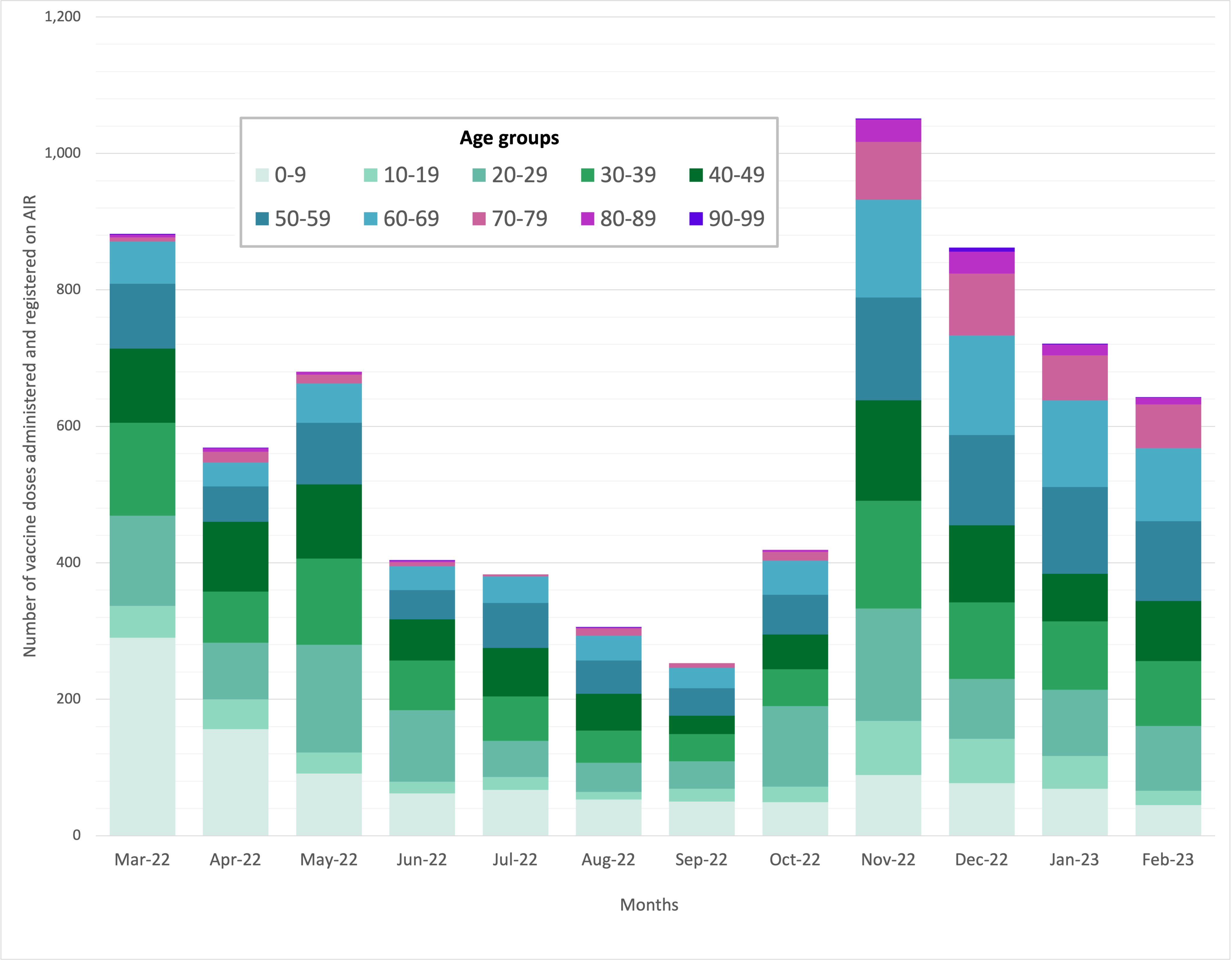

Supplement: Misan et al. supplementary material [file S0950268824001730sup001.zip › SF 1.png]
